# Investigation of Melts of Polybutylcarbosilane Dendrimers by ^1^H NMR Spectroscopy

**DOI:** 10.1038/s41598-017-13743-z

**Published:** 2017-10-20

**Authors:** Vladimir V. Matveev, Denis A. Markelov, Sergey V. Dvinskikh, Andrei N. Shishkin, Konstantin V. Tyutyukin, Anastasia V. Penkova, Elena A. Tatarinova, Galina M. Ignat’eva, Sergey A. Milenin

**Affiliations:** 1 St. Petersburg State University, 7/9 Universitetskaya nab., St. Petersburg, 199034 Russia; 2 St. Petersburg National Research University of Information Technologies, Mechanics and Optics, Kronverkskiy pr. 49, St. Petersburg, 197101 Russia; 3 Department of Chemistry, Royal Institute of Technology KTH, Stockholm, SE 10044 Sweden; 4 Enikolopov Institute of Synthetic Polymeric Materials, Russian Academy of Sciences, 70 Profsoyuznaya St., 117393 Moscow, Russia

## Abstract

Melts of polybutylcarbosilane (PBC) dendrimers from third (G3) up to sixth (G6) generations are investigated by ^1^H NMR spectroscopy in a wide temperature range up to 493 K. At room temperature, NMR spectra of G3-G5 dendrimers exhibit resolved, solution-like spectra (“liquid” phase). In contrast, the spectrum of the G6 dendrimer is characterized by a single unresolved broad line at whole temperature range, which supports the presence of an anomalous phase state of G6 at temperatures higher than glass transition temperature. For the first time, an unexpected transition of G5 dendrimer from a molecular liquid state to an anomalous state/phase upon *temperature increase* has been detected using NMR data. Specifically, an additional wide background line appears in the G5 spectrum above 473 K, and this line corresponds to a G5 state characterized by restricted molecular mobility, i.e., a state similar to the “anomalous” phase of G6 melt. The fraction of the G5 dendrimers in “anomalous” phase at 493 K is approximately 40%. Analysis of the spectral shapes suggests that changes in the G5 dendrimers are reversible with temperature.

## Introduction

Dendrimers are unique macromolecules with regular, treelike architecture^1,2^. Stepwise cascade synthesis allows one to obtain macromolecules with monodisperse distribution of size and structure. The chemical composition of the dendrimers can be very flexible, which allows the possibility of synthesizing macromolecules with specific properties targeted for various applications in polymer chemistry, biology and medicine^3–6^.

Melts of dendrimers with different structures, for example, poly(amido amine) (PAMAM) dendrimers^7–11^, poly(propylene imine) (PPI) dendrimers^12–14^, carbosilane dendrimers^15–27^, were investigated above and below the glass transition temperature using various experimental techniques. In this paper we study melts of polybutylcarbosilane (PBC) dendrimers of several generations: from third (G3) to sixth (G6). Past studies have found a difference in behavior between higher and lower generation dendrimers. This difference was first observed in ref.^15^. The thermodynamic properties of a melt of PBC G6 dendrimers differ sharply from those of dendrimers with *G* < 6. In the temperature dependence of the heat capacity, *C*_*p*_, two transition ranges were observed for the G6 dendrimer compared to one transition as *G* < 6 (see Fig. S1 in Supplementary Information (SI)). Authors of ref.^15^ established that the low temperature increase corresponded to the glass – melt transition. The glass temperature, *T*_*g*_ is practically independent of dendrimer generations of the same homologous row (see Table 1). In the high temperature region of the *C*_*p*_ temperature dependence a significant increase in growth rate (370–460 K) was observed for G6 dendrimer. In the case of G5 dendrimer, a similar effect was absent, but an anomaly (i.e. a non-monotonic behavior) was also observed (see Fig. S1 in SI).

**Table 1:** Structural parameters of the PBC dendrimers. N_i_ is a portion of hydrogen atoms contributing to the signal of the peak #i to the total number of hydrogen atoms in the dendrimer at room temperature; T_g_ is glass transition temperature (data from ref.^15^).

G	*Molecular weight* a.u.m.	*Chemical formula*	*N*_*i*_ *(Theory)/N*_*i*_ *(Experimental)*				*T*_*g*_ *K*
			Peak 1 CH_3_-Si	Peak 2 -CH_2_-Si	Peak 3 CH_3_-	Peak 4 -CH_2_-	
3	4232	Si_29_C_240_H_540_	0.16/0.16	0.32/0.30	0.18/0.18	0.34/0.36	179.8
4	8776	Si_61_C_496_H_1116_	0.16/0.15	0.33/0.32	0.17/0.19	0.34/0.34	186.0
5	17864	Si_125_C_1008_H_2268_	0.16/0.18	0.33/0.30	0.17/0.19	0.34/0.33	186.5
6	36040	Si_253_C_2032_H_4572_	0.17	0.33	0.17	0.33	186.2

The phase between these two *C*_*p*_ features was studied by different experimental methods^15,17–20,28,29^. It has been suggested that the anomalous phase is a consequence of the physical entanglements in melts of high generation dendrimers^19,28^. Moreover, the study of rheological properties led authors of ref.^18^ to conclude that high generation carbosilane dendrimers formed a supramolecular structure through a physical network of intermolecular contacts. The presence of physical entanglements was supported by atomistic molecular dynamics simulations of dendrimer melts in recent theoretical studies^30,31^.

However, the mechanism of the physical entanglements in the PBC dendrimer melts is still unclear. At this point, two hypotheses about the nature of the entanglements have been published. According to one hypothesis^31^ low and high density regions are formed in the peripheral layer. The formation of these regions is associated with steric constraints arising from the growth of dendrimer generations. Authors of work^31^ suppose that high density regions lead to entanglements between dendrimers. An alternative hypothesis^30^ is that intermolecular cavities grow with increasing *G* due to the increase of the dendrimer size and the decrease in the penetration of macromolecules into each other. These factors may lead to the formation of a compact structure that minimizes free space in such systems^32^.

Additionally, we would like to underline that the presence of entanglements in the melt of hyperbranched polymers is experimentally established in ref.^33^.

To the best of our knowledge, this effect has not been investigated by NMR spectroscopy, which is one of the most efficient tools to study structure, conformations and dynamics of macromolecules^34–37^. Also note that NMR spectroscopy has been successfully applied to studies of dendrimers in solutions in our works^38–40^. In this paper we apply ^1^H NMR spectroscopy to reach new insights on the molecular dynamics and phase transitions of PBC dendrimers.

## Experimental

**Figure 1:**
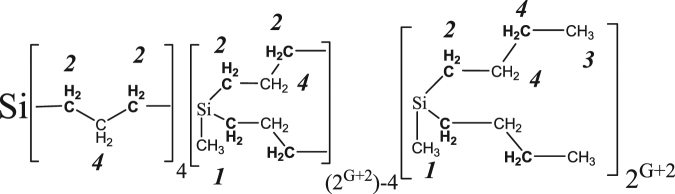
The structure of polybutylcarbosilane (PBC) dendrimers of G = 3–6 generations. Numbers indicate assignments of the structural groups to the peaks in the ^1^H NMR spectra (Fig. 2).

The structure of the PBC dendrimers is shown in Fig. 1 and some parameters of the dendrimers are presented in Table 1. In the studied PBC dendrimer, the core functionality was 4 and the functionality of the branching points was 3. The synthesis of PBC dendrimers was described earlier^16^. The structure and composition of the PBC dendrimers was confirmed by the ^1^H NMR spectra (see Table 1). The measurements were carried out at 500 MHz using a BRUKER AVANCE 500 spectrometer. The chemical shifts, *δ*, were referenced by setting the position of peak 1 to −0.05 ppm according to data for carbosilane dendrimers in chloroform^38^. The PBC dendrimer melts were studied in the range of 298–493 K, which was significantly higher than *T*_*g*_. For each temperature point, the equilibration time was at least 20 minutes. The number of scans varied between 8 and 32 depending on the initial signal-to-noise ratio in the NMR spectrum. The integral of each spectrum was normalized by the number of protons in accordance with the theoretical formula of the dendrimer (see Table 1). ACD/Labs software was applied in the deconvolution of G5 experimental spectrum into a number of lorentzian components, using the Levenberg-Marquardt algorithm.

For diffusion measurements, the pulsed-field-gradient stimulated echo method was used with maximum gradient strength 1280 G/cm. The gradient pulse length and the diffusion time were set to 4 ms and 400 ms, respectively. The proton spin-lattice relaxation times, *T*_1H_, were measured using conventional inversion-recovery *π*-*τ*-*π*/2 pulse sequence with 6 μs duration of *π*/2 pulse, 16 tau delays, 2 scans for each delay, and a 3 to 8 s recycle time between scans. In the paper we use relaxation rate function *R*_1H_ = 1/*T*_1H_. The proton spin-spin relaxation was measured by a spin-echo sequence *π/2*-*τ*-*π*/2, 16 tau delays, 8 scans for each delay, and a 3 to 8 s recycle time between scans.

## Results

**Figure 2:**
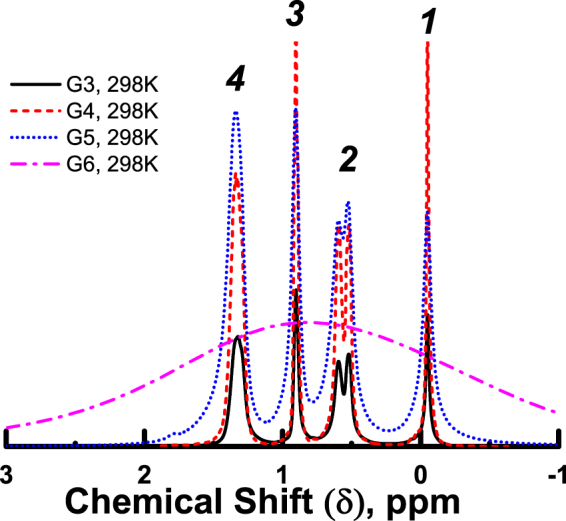
^1^H NMR spectra of G3-G6 PBC dendrimer melt at 298 K. The peak assignment (1–4) is shown in Fig. 1 and Table 1.

Room temperature NMR spectra of PBC dendrimers of G3-G6 generations are displayed in Fig. 2. Spectra of G3-G5 exhibit resolved peaks that were assigned to different structural groups. The spectra can be divided into four main groups of peaks in accordance with Table 1. Peak with chemical shift (*δ)* near zero ppm corresponds to the signal of the СН_3_-Si group with a chemical environment similar to that found in the reference compound Si(CH_3_)_4_. The following peaks, in ascending order of *δ*, are СН_2_-Si, terminal СН_3_-groups neighboring with СН_2_-groups, and finally the СН_2_-groups, which are neighbors of СН_2_- or СН_3_-.

In contrast, a spectrum of G6 melt consists of a single, unresolved broad line covering the entire area of expected chemical shifts. Such a shape of the spectrum can be explained as a consequence of much higher viscosity of the G6 melt compared to that of G3-G5 dendrimers. This high viscosity reduces the *T*_2_^*^ relaxation time of each spectral line, leads to a significant increase of the line width, Δν = 1/(*πT*_2_^*^)^41^, and results in the unresolved total G6 spectrum.

In order to obtain a temperature dependence of the G6 linewidth it is necessary to keep in mind that the unresolved chemical shift range contributed to the observable linewidth of total spectrum.

**Figure 3:**
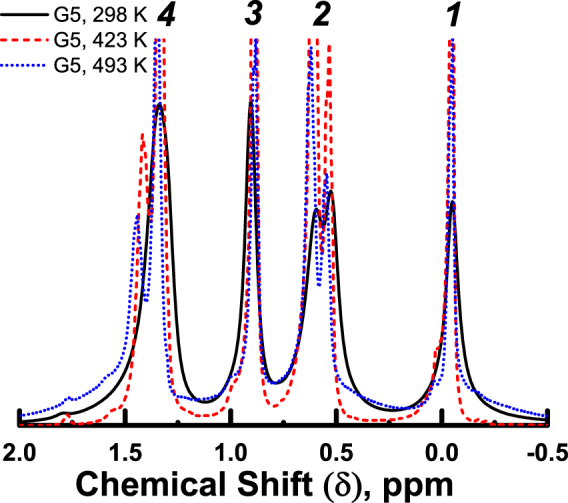
Temperature dependent spectra of the G5 dendrimer. The peak assignment (1–4) is shown in Fig. 1 and Table 1.

**Figure 4:**
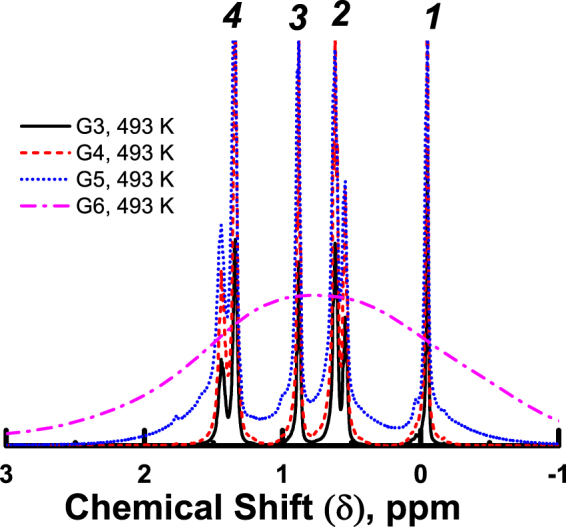
^1^H NMR spectra of G3-G6 PBC dendrimer melt at 493 K. The peak assignment (1–4) is shown in Fig. 1 and Table 1.

G3 and G4 samples display relatively narrow lines, whose widths continuously decrease with increasing temperature. In contrast, the G5 sample exhibits complex behavior of the spectrum in different temperature ranges (see Fig. 3). A conventional narrowing of peak width and an increase of spectral resolution with increasing temperature are observed between 298 and 423 K. However, an additional broad background peak appears in the spectra at 473 K and higher temperatures (see Fig. 3 and SI for more details). We would like to emphasize that no broadening is observed for narrow spectral lines, and, hence, it is not possible to fit the shape of whole G5 spectrum at 473 K without adding an additional background line. We have verified that no similar effect is present in G3 and G4 dendrimers (Fig. 4).

**Figure 5:**
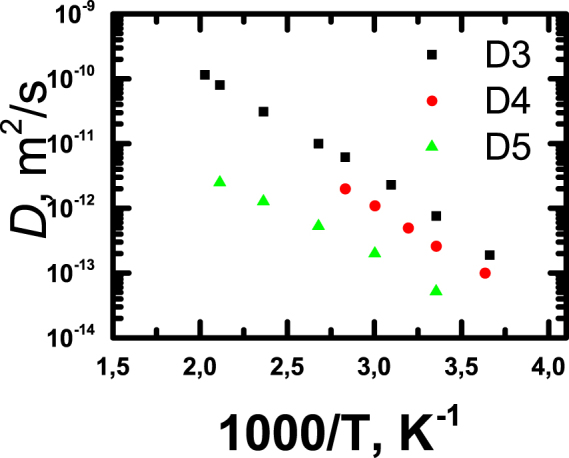
Temperature dependence of diffusion coefficients of G3-G5 dendrimers in melt.

**Figure 6:**
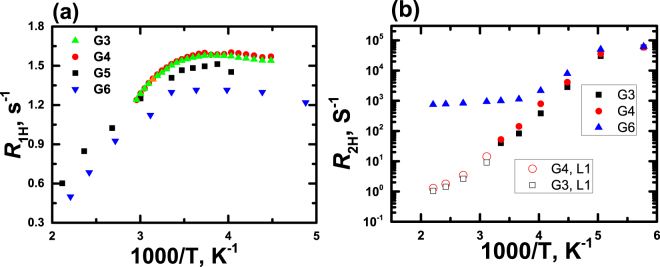
Temperature dependences of spin-lattice (**a**) and spin-spin (**b**) relaxation rates of PBC dendrimers.

The diffusion coefficients *D* for *G* = 3–5 dendrimers were measured at different temperatures and, as expected, decrease with both an increase in molecular mass and a decrease in temperature (Fig. 5). Unfortunately, we could not measure *D* for G6 even at elevated temperatures, due to the limitation imposed by a very short spin-spin relaxation time *T*_2_. The difference in spin-spin relaxation rate *R*_2H_ = 1/*T*_2H_ between G6 and other dendrimers can be seen in Fig. 6b.

In contrast to large differences in the shapes and widths of the NMR spectra, differences in NMR spin-lattice relaxation rates *R*_1H_ of the G3-G6 dendrimers are not so significant (Fig. 6a). First of all, numerical values of measured *R*_1H_ are of the same order of magnitude and this means that the local orientational mobility exists in all dendrimers, including in G6. Moreover, in the investigated temperature range, broad *R*_1H_ maxima were found for all investigated dendrimers with *R*_1H_ maximum positions which are not far from one another, see Fig. 6a. According to the theory of orientation mobility in dendrimers^42–44^ these results suggest that local orientational mobility in G6 is similar to the mobility of low generation dendrimers.

**Figure 7:**
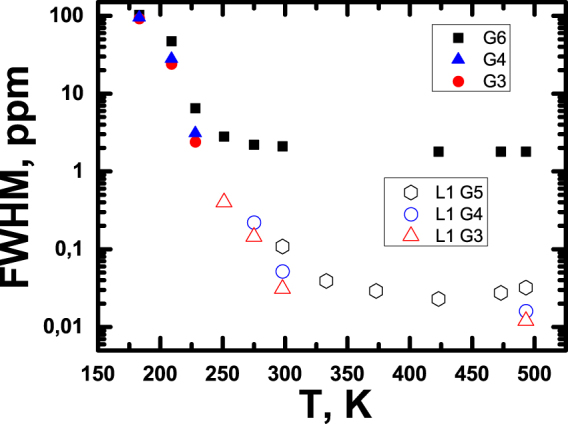
Full width at half maximum (FWHM) of lines of PBC dendrimer for unresolved spectra and line #1 (at −0.05 ppm) for other spectra.

In addition, in Fig. 7 we show the full width at half maximum (FWHM) of lines of PBC dendrimers for unresolved spectra and line #1 (at −0.05 ppm) for other spectra.

## Discussion

As mentioned above, sample G6 is characterized by one wide spectral line. This fact may be associated with the anomalous phase, which has been observed in previous studies by other methods^15,18,19,28^. Since no anomalous phase is present in low generation dendrimers (<G5), highly resolved spectra are observed (Fig. 2). We will refer to such a low generations phase as an ordinary “liquid” phase.

**Figure 8:**
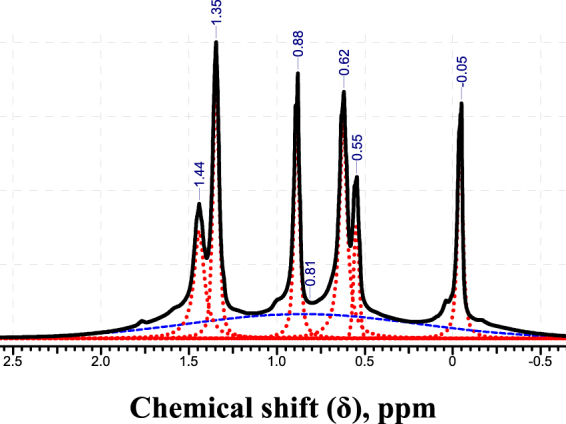
The ^1^Н NMR spectrum of G5 dendrimer melt at 493 K (solid black peak) and the decomposition of the spectrum into components: red dotted peaks show the fitting of standard narrow peaks; blue dashed peak marks the wide peak, which is similar to the G6 spectrum.

Now we would like to discuss the anomalous temperature behavior of the spectra of G5 dendrimer melt (Fig. 3). In order to fit G5-melt spectrum at 493 K correctly, we have to introduce an additional broad component (line #5). We assume that the shape of the broad line is similar to that of the unresolved G6 spectrum. Thus, to fit this line we used the same parameter of Lorentz/Gauss ratio which gave the best fit for G6 PBC dendrimer spectrum (see Fig. S5 in SI). Deconvoluted G5-melt spectrum at 493 K is shown in Fig. 8.

The relative integral intensities of the spectral components are summarized in Table 2. As seen in Tables 1 and 2, the relative integral intensities of the narrow peaks 1–4 (i.e., excluding the additional broad peak which is marked #5 in Table 2) correspond to the ratios calculated using a structural formula of the dendrimer. This correspondence indicates that the appearance of the broad component (peak 5) is associated with changes in the dynamics of the dendrimer as a whole but not with changes in the mobility of some fragments of the macromolecule. The shape and position of peak 5 are indeed similar to the spectrum of G6 melt (see Fig. S5 in SI). This suggests that a part of the G5 dendrimer is in an anomalous phase similar to the melt of G6. A fraction of this part of the G5 can be estimated by comparing integral intensities of the broad peak 5 and of the remaining narrow peaks 1–4 in the spectrum. As shown in Table 2, a fraction of the second phase at 493 K is ca. 42% ± 4%. We note that the peak 5 disappears when returning the sample to a temperature of 423 K, i.e. the formation of the second phase is reversible.

**Table 2:** Spectrum deconvolution of G5 dendrimer melt at 493 K. The peak numbering corresponds to an increase in their chemical shift.

Peak #	Chemical shift (δ), ppm	Integral Intensity (% of the total integral)	Integral Intensity (% of the total integral without integral of peak #5)	Theoretical Integral intensity (% of the total integral without integral of peak #5)
1	−0.05	9	15	16
2a	0.55	4	32	33
2b	0.62	14		
3	0.88	9	16	17
4a	1.35	12	37	34
4b	1.44	10		
5	—	42	—	—

It is important to note that the physical processes, leading to the temperature dependent changes in the spectral shapes of the G5 sample at low temperature and in the vicinity of 470–490 K, are not the same. The conventional broadening of the spectral peaks of macromolecules at lowering temperature is caused by a decrease in molecular mobility and a related decrease of spin-spin relaxation times *T*_2_^41^. This broadening is observed for dendrimers of low generations (see Figs 2 and 4). On the contrary, the additional broad peak in the spectrum of G5 dendrimer, emerging at high temperatures, is associated with a part of the sample undergoing phase transition to an anomalous state, while the rest of the sample exhibits narrow peaks typical for conventional liquids. As an illustration, the temperature dependences of the full widths at half maximum (FWHM) of line #1 are included in Fig. 7.

We would like to stress the coincidence between the region of the nonmonotonic behavior in *C*_*p*_ (370 K–460 K, see Fig. S1 in SI) and the appearance of the broad line of spectra for G5 PBC dendrimers. The spectra at 473 K and 493 K where we can reliably determine the broad line are immediately above the maximal temperature of this region. This suggests that the nonmonotonic behavior in the *C*_*p*_ reflects the formation of a new state in the G5 melt. Given the similarity of the NMR spectral shapes, it is reasonable to assume that the new phase in G5 is analogous to the G6 anomalous phase. Thus, besides unexpected temperature changes in NMR spectral shapes, our results demonstrate a rather unusual effect – a transition of a part of the G5 dendrimers from the “liquid” state to the anomalous state *upon temperature increase*. The simultaneous presence in the NMR spectrum of a broad unresolved peak, corresponding to an anomalous phase, and a set of relatively narrow peaks, corresponding to the “liquid” state of dendrimer, indicates the coexistence of the two states.

Thus, the results of this work indicate a similarity in the behavior of G5 and G6 dendrimers, which manifests in the formation of aggregates of dendrimer molecules in an anomalous state. In the G6 melt such clusters cover all or at least an overwhelming part of the melt, while in the G5 melt the clusters appear only at 473 K and above and coexist with the molecular state of the dendrimer.

## Electronic supplementary material

SUPPLEMENTARY INFORMATION
