# Supplementary material for: Investigation of Melts of Polybutylcarbosilane Dendrimers by 1H NMR Spectroscopy
Source: Sci Rep. 2017 Oct 20;7:13710. doi: 10.1038/s41598-017-13743-z (PMC5651846; doi:10.1038/s41598-017-13743-z)
Supplement: Supplementary file 1 — SUPPLEMENTARY INFORMATION [file 41598_2017_13743_MOESM1_ESM.pdf]

**SUPPLEMENTARY INFORMATION**

# Investigation of Melts of Polybutylcarbosilane Dendrimers by $^1$H NMR Spectroscopy

*Vladimir V. Matveev,* [a] *Denis A. Markelov,* [a,b] *Sergey V. Dvinskikh,* [a,c] *Andrei N. Shishkin,* [a] *Konstantin V. Tyutyukin,* [a] *Anastasia V. Penkova,* [a] *Elena A. Tatarinova,* [d] *Galina M. Ignat'eva,* [d] *Sergey A. Milenin* [d]

[a] *St. Petersburg State University, 7/9 Universitetskaya nab., St. Petersburg, 199034, Russia*

[b] *St. Petersburg National Research University of Information Technologies, Mechanics and Optics, Kronverkskiy pr. 49, St. Petersburg, 197101, Russia.*

[c] *Department of Chemistry, Royal Institute of Technology KTH, Stockholm, SE 10044, Sweden*

[d] *Enikolopov Institute of Synthetic Polymeric Materials, Russian Academy of Sciences, 70 Profsoyuznaya St., 117393 Moscow, Russia*

## Heat capacity for PBC dendrimers melt.

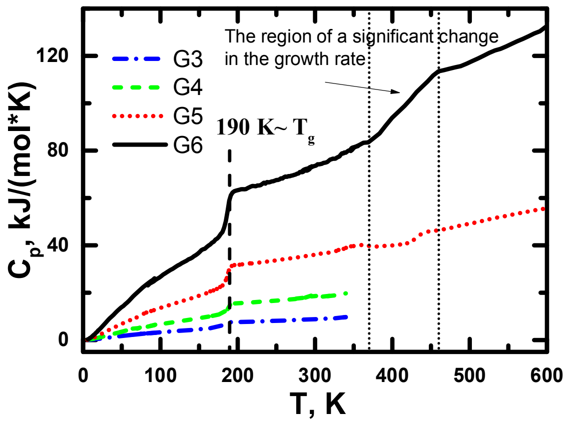


**Figure S1.** *Temperature dependence of heat capacity for polybutylcarbosilane (PBC) dendrimers by using data obtained from Ref.*[15]

## Temperature Evolution of $^1H$ Spectra of PBC dendrimer G5 melt

The increase of melt temperature from 298 K to 423 K leads to narrowing peaks in NMR spectrum of G5 PBC dendrimer (Fig. S2). This effect is natural for polymer and liquid systems and corresponds to the increase in segmental mobility of a macromolecule. Further heating results in the appearance of an additional broad peak, but after cooling the peak disappears (Fig. S3). No difference between spectra at 473K and at 493 K is practically observed.

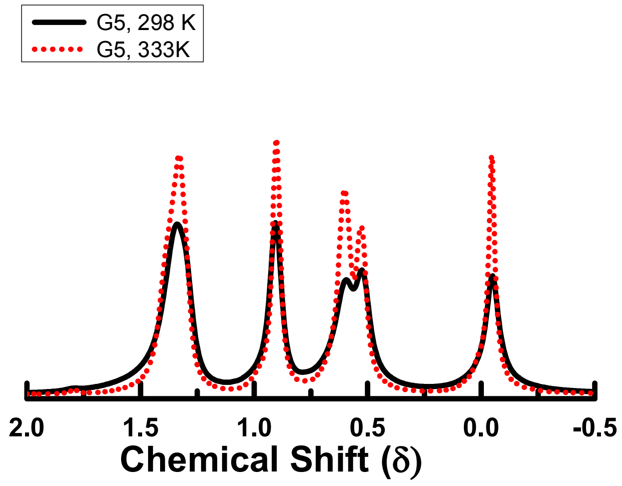

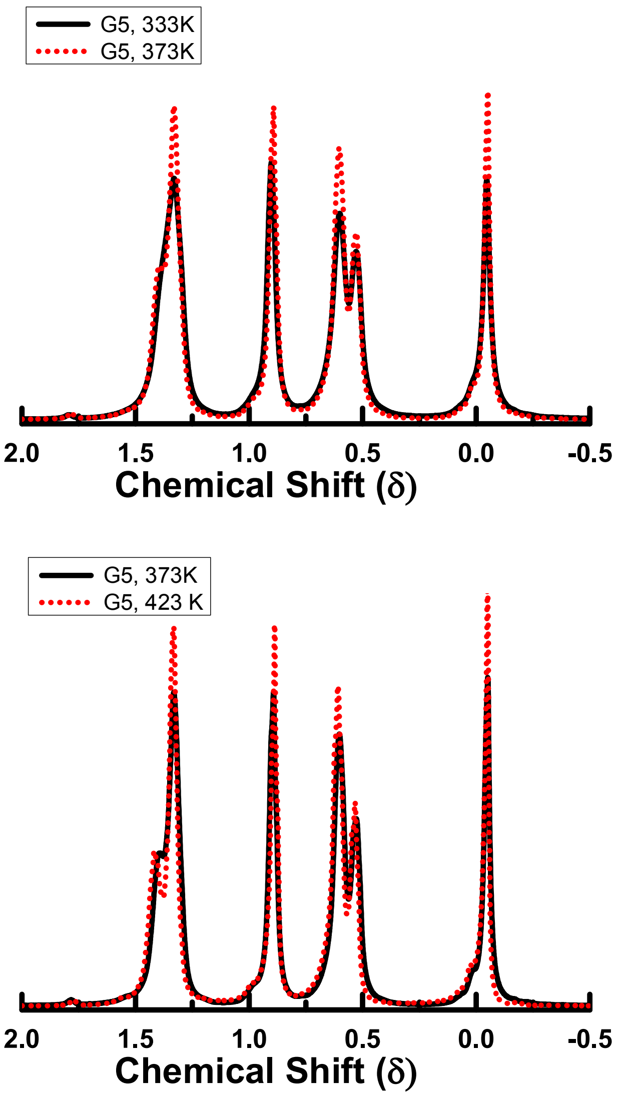


**Figure S2.** *Changes of $^1$H-NMR spectrum of G5 PBC dendrimer melt from 298 K to 423 K.*

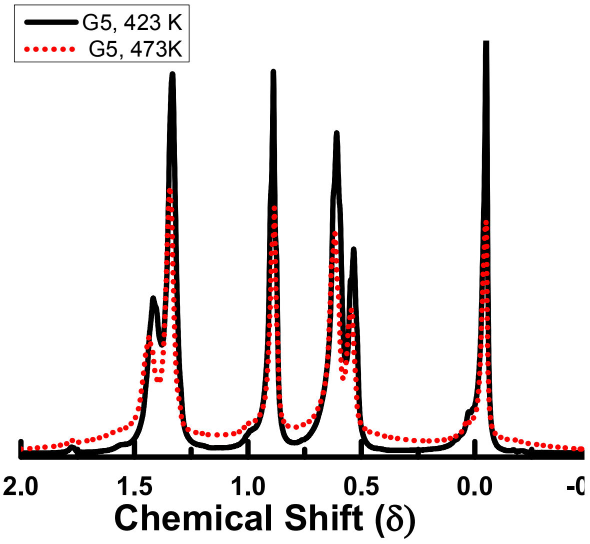

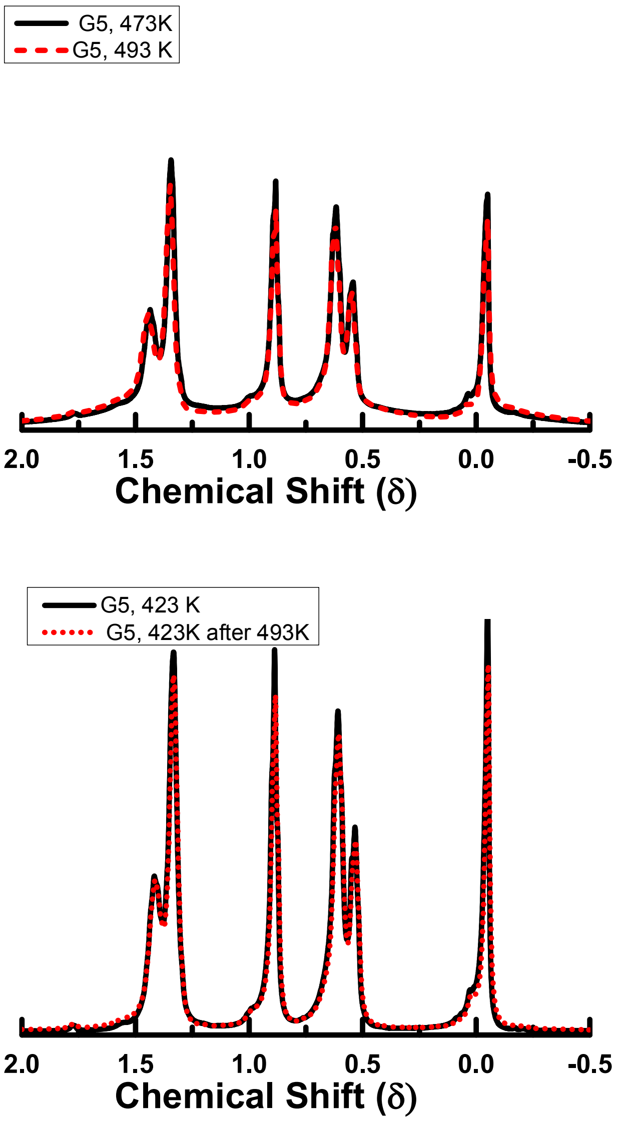


**Figure S3.** $^{1}$*H-NMR spectrum of G5 PBC dendrimer melt at 423 K, 473 K, 493 K , and at 423 K after heating up to 493 K.*

## Temperature Evolution of $^{1}$H Spectra of PBC dendrimer melts at low temperatures

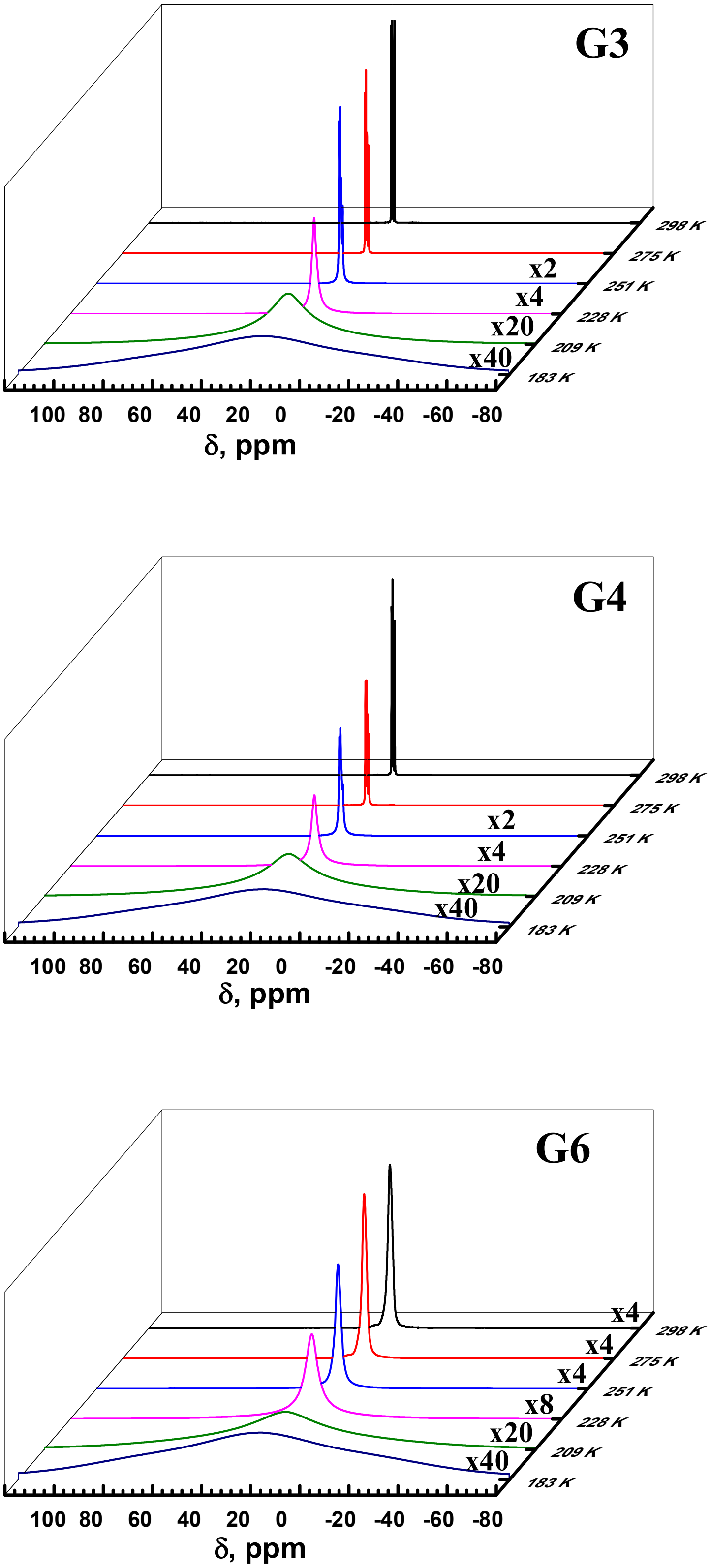


**Figure S4.** *Temperature dependence of $^{1}$H-NMR spectrum of the melt of PBC dendrimers G3, G4, G6.*

## Fitting of Broad Lines in G6 and G5 spectra at 493K

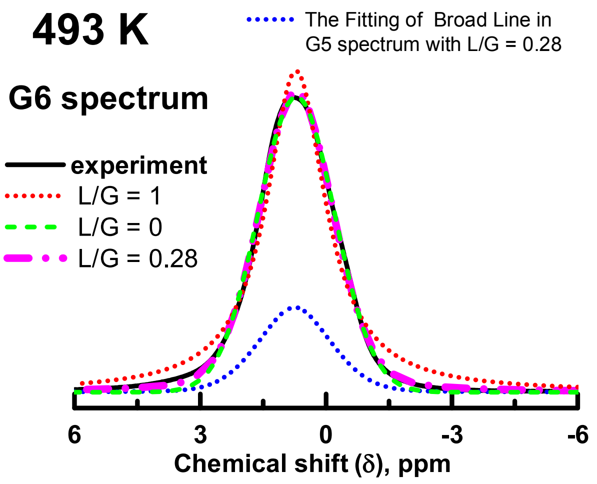


**Figure S5.** *$^{1}$H-NMR spectrum of the melt of G6 dendrimers at 493 K and its fitting by Lorentz (L/G = 1), Gauss (L/G = 0), and combined Lorentz / Gauss equations. The L/G = 0.28 for the combined line was chosen as this value gave the best fit for the line. Also is shown the broad line of G5 spectrum extracted at 493 K using the same fitting procedure with L/G = 0.28.*
